# Effects of Thermal Aging on Molar Mass of Ultra-High Molar Mass Polyethylene Fibers

**DOI:** 10.3390/polym14071324

**Published:** 2022-03-24

**Authors:** Zois Tsinas, Sara V. Orski, Viviana R. C. Bentley, Lorelis Gonzalez Lopez, Mohamad Al-Sheikhly, Amanda L. Forster

**Affiliations:** 1 Material Measurement Laboratory, National Institute of Standards and Technology, Gaithersburg, MD 20899, USA; zois.tsinas@nist.gov (Z.T.); sara.orski@nist.gov (S.V.O.); viviana.rodriguezcardenas@nist.gov (V.R.C.B.); 2 Theiss Research, La Jolla, CA 92037, USA; 3 Materials Science and Engineering Department, University of Maryland, College Park, MD 20742, USA; lorelis@terpmail.umd.edu (L.G.L.); mohamad@umd.edu (M.A.-S.)

**Keywords:** ultra-high molar mass polyethylene (UHMMPE), high strength fiber, molar mass, thermal aging

## Abstract

Ultra-high molar mass polyethylene (UHMMPE) is commonly used for ballistic-resistant body armor applications due to the superior strength of the fibers fabricated from this material combined with its low density. However, polymeric materials are susceptible to thermally induced degradation during storage and use, which can reduce the high strength of these fibers, and, thus, negatively impact their ballistic resistance. The objective of this work is to advance the field of lightweight and soft UHMMPE inserts used in various types of ballistic resistant-body armor via elucidating the mechanisms of chemical degradation and evaluating this chemical degradation, as well as the corresponding physical changes, of the UHMMPE fibers upon thermal aging. This is the first comprehensive study on thermally aged UHMMPE fibers that measures their decrease in the average molar mass via high-temperature size exclusion chromatography (HT-SEC) analysis. The decrease in the molar mass was further supported by the presence of carbon-centered free radicals in the polyethylene that was detected using electron paramagnetic resonance (EPR) spectroscopy. These carbon-centered radicals result from a cascade of thermo-oxidative reactions that ultimately induce C–C ruptures along the backbone of the polymer. Changes in the crystalline morphology of the UHMMPE fibers were also observed through wide-angle X-ray diffraction (WAXS), showing an increase in the amorphous regions, which promotes oxygen diffusion into the material, specifically through these areas. This increase in the amorphous fraction of the highly oriented polyethylene fibers has a synergistic effect with the thermo-oxidative degradation processes and contributes significantly to the decrease in their molar mass.

## 1. Introduction

Many types of ballistic-resistant body armor, including soft armor, rifle-protective plates, and helmets rely upon the ultra-high molar mass polyethylene (UHMMPE) fiber, which is comprised of very well-aligned (drawn 50 to 100 times the original length during processing), highly crystalline (above 85%), very high molar mass polymer chains, with a molar mass range between 3 and 5 million g/mol [1,2,3,4]. Given the large amount of heavy equipment that must be carried by law enforcement and military personnel, designing armor that combines the highest possible protection with the lightest weight is critical for the health and safety of armor wearers [5,6,7]. UHMMPE fibers offer a unique combination of high strength (up to 4 GPa [8]) combined with a low density (0.97 g/cm^3^) [8,9], resulting in lighter weight armor. Armor users expect their armor to continue to protect them for the duration of its service life (typically 5 years) [10]. In response to a field failure in 2003, which was attributed to the hydrolysis of a body armor made from the fiber poly(*p*-phenylene-2,6-benzobisoxazole), many efforts have been directed toward understanding the long-term stability of other classes of fibers used in body armor when exposed to environmental conditions [11,12,13,14,15,16].

Polyolefins are generally expected to be most sensitive to degradation due to oxidation [17,18]. The oxidation can proceed from defects, impurities, and unsaturations that are created in the polymer during processing, which eventually results in the formation of hydroperoxides [17]. Prior work, mostly focused on UHMMPE for use in orthopedic applications, fully describes the mechanism and rates of oxidation-induced degradation in polyethylene [17,18,19,20,21]. Some environmental factors are not relevant for ballistic-resistant articles. For example, photo-oxidation is generally prevented for most types of body armor by a protective carrier or covering for the armor, which prevents ultraviolet light exposure [22]. Probable exposure conditions for armor include mechanical damage due to wear, namely folding and abrasion, combined with elevated temperature and humidity. Mechanical degradation can initiate oxidation through a process known as mechano-oxidation [17], and UHMMPE fibers have been shown to be more susceptible to oxidize at the mechanically damaged or kink-banded areas of the fiber [23]. Oxidation-induced degradation of polyolefins may lead to chain scission, causing a reduction in the molar mass of the polymer [24,25,26,27,28]. Given that the high strength of UHMMPE fibers originates from their molecular structure of long, highly aligned chains of carbon–carbon bonds (molar masses of this system are reportedly between 3 million and 5 million g/mol), chain scission is expected to have a detrimental effect on its mechanical properties [25,29]. 

The effects of thermal degradation are also critical for the long-term stability of polyolefins because organic molecules have been previously shown to be unstable and can depolymerize above certain temperatures [30]. More specifically, the decomposition temperature of high-density polyethylene (HDPE) is around 400 °C [31]. Typically, the bond dissociation enthalpies of C–C and C–H bonds at 25 °C are reported to be within the range of 260 kJ/mol to 400 kJ/mol and 320 kJ/mol to 420 kJ/mol, respectively [30]. When energy greater than the mean energy of these bonds is transferred to an UHMMPE molecule, scission of these bonds can occur. In condensed systems, such as the highly oriented and very crystalline polyethylene fibers studied here, the vibrational energy from heat is rapidly dissipated among all molecules and bonds leading to a highly excited vibrational state of the bonds, which could induce a high repulsive energy level and cause bond rupture of C–C and/or C–H bonds, even at much lower temperatures [30]. Molecular dynamics modeling studies are also in support of C–C bond dissociation in polyethylene with much lower activation energies (in the order of 40 kJ/mol to 190 kJ/mol) than the above typical values [32]. Additionally, similar studies have previously shown that the activation energy of the C–C bond scission in the backbone of polymers, and polyethylene more specifically, decreases as molar mass increases [33,34]. This is particularly relevant to our work, since the UHMMPE fibers studied here have molar masses on the order of millions of g/mol. These bond scission events result in the formation of carbon-centered radicals in polyethylene, namely alkyl free radicals. In the presence of oxygen, mainly in the amorphous phase of the UHMMPE fibers, alkyl free radicals participate in a series of reactions that can further decrease the molar mass of the polymer through oxidative degradation [23]. 

Prior work examined the long-term stability of UHMMPE fibers, using both oxygen uptake [35] and tensile strength [16] as the degradation metrics, and determining the activation energy for this process to be between 120 kJ/mol and 140 kJ/mol. The effect of aging on the mechanical properties of UHMMPE was thoroughly examined, and oxidation was characterized in the system using Fourier Transform Infrared (FTIR) spectroscopy and the computation of the oxidation index. However, while the reduction in tensile strength was surmised to be attributed to oxidative chain scission, no accessible methods to investigate changes in molar mass in the aged material were available at the time of publication of that study. This work will describe for the first time a method for performing high-temperature size exclusion chromatography (HT-SEC) on UHMMPE fibers and present the effect of thermal aging on the molar mass distribution of this material. To further support the decrease in molar mass and the rupture of C–C bonds from the backbone of UHMMPE, electron paramagnetic resonance will be used to detect the presence of carbon centered radicals in the aged specimens. Finally, the crystallinity of the material after thermal aging will be examined using wide angle X-ray scattering (WAXS) to fully investigate the effects of thermal aging and chain scission on the crystal structure and total crystallinity of this material. 

## 2. Materials and Methods

### 2.1. Aging Experiments

Gel spun UHMMPE continuous filament yarns were used in this study. Yarn samples were stored in dark ambient conditions prior to and after aging. For the aging experiments, yarns were wound using a custom-designed fiber-winding device onto perforated spools and placed into ovens at 43 °C, 65 °C, 90 °C, and 115 °C under dry conditions for a predetermined period of time of 81 w, 43 w, 17 w, and 8 w, respectively. The ovens used for the thermal aging of the fibers are ESPEC, model BTL-433. The air in the ovens was circulating and maintained at the desired temperature. The temperatures were specifically selected to accelerate the thermal aging of the materials in order to achieve a realistic experimental time. Lower temperatures of 43 °C and 65 °C were selected to assess thermal degradation closer to the relevant body temperature at 37 °C. The higher temperatures of 90 °C and 115 °C aim to identify thermal degradation phenomena and mechanisms that occur around the alpha relaxation of the material at about 80 °C, or above that, respectively. A series of temperature and relative humidity dataloggers were used to monitor the consistency of the chamber temperature during the exposure period. Full details of the study are given in a prior publication [16].

### 2.2. Molar Mass Distribution Determination

High-temperature size exclusion chromatography (HT-SEC) was used to determine the molar mass distribution. Samples were prepared based on an adaptation of a method developed for another effort [36,37]. Polyethylene yarn samples were encased in a 5 µm stainless-steel mesh packet and dissolved in HPLC grade 1,2,4-trichlorobenzene (TCB, under nitrogen atmosphere) at around 160 °C and with continuous shaking for a minimum of 2 h before injecting. Approximately 10 mg of antioxidant (Irganox 1010) was added to each vial during dissolution. The mesh packets were removed, and the solutions were then transferred to the high-temperature autosampler (at approximately 160 °C).

These dissolved yarn samples were analyzed by HT-SEC using a Tosoh (The full description of the procedures used in this paper requires the identification of certain commercial products and their suppliers. The inclusion of such information should in no way be construed as indicating that such products or suppliers are endorsed by NIST or are recommended by NIST or that they are necessarily the best materials, instruments, software, or suppliers for the purposes described) HT-EcoSEC instrument with differential refractive index (RI) detection. Separations were conducted at approximately 160 °C using 1,2,4-trichlorobenzene as the eluent, with nominally 300 mg/kg Irganox 1010 added as antioxidant to the solvent reservoir. About 5 mL of dodecane were added to each vial as a flow rate marker. The stationary phase was a set of 3 Tosoh HT columns (2 Tosoh TSKgel GMHHR-H (S) HT2, 13 μm mixed bed, 7.8 mm ID × 30 cm columns and 1 Tosoh TSKgel GMHHR-H (20) HT2, 20 μm, 7.8 mm ID × 30 cm column with an exclusion limit ≈ 4 × 10^8^ g/mol). Narrow dispersity polystyrene standards were used for calibration. The resulting universal calibration curve, the plot of Log ([n]*M) as a function of retention volume, was determined through the Mark–Houwink equation (Equation (1) below), where K and α are known empirical Mark–Houwink constants at 160 °C for PS and PE in TCB. The values used are the ones recommended by ASTM D 6474 at these conditions (K_PS_ = 1.21 × 10^−4^ dL/g; α_PS_ = 0.707; K_PE_ = 4.06 × 10^−4^ dL/g; α_PE_ = 0.725) [38].

[η] = KM^α^ (1)

where [η] is the intrinsic viscosity and M is the molecular weight. 

The uncertainty in the molar mass precision obtained by this measurement is ±1.5%. All injections were performed at least three times, and the reported error on all measurements is one standard deviation of the mean.

### 2.3. Crystallinity Determination and Wide-Angle X-ray Scattering

Wide angle X-ray scattering (WAXS) measurements were conducted using a Xenocs Xeuss SAXS/WAXS small angle X-ray scattering system (Xenocs, Grenoble, France). The instrument was equipped with an X-ray video-rate imager detector for WAXS analysis (up to about 45° 2θ). All diffractograms were collected at room temperature [29]. The incident beam, diffracted beam and sample chamber were kept under vacuum (below 0.1 mbar). The bundle of UHMMPE fibers was mounted horizontally, perpendicular to the direction of the X-ray beam. Silver behenate was used as a control and each sample was tested in duplicate [39].

### 2.4. Fiber Topography and Chemical Composition

The shape and the surface morphology of the aged UHMMPE fibers were characterized by scanning electron microscopy (SEM). A Hitachi S-2400 (Hitachi, Tokyo, Japan) variable pressure SEM equipped with an X-ray detector that allows for elemental analysis through energy dispersive spectroscopy (EDS) was used. The fibers were evenly spread over slabs and left under vacuum overnight to eliminate any traces of humidity and oxygen bound to the surface of the fibers prior to analysis. The analysis was conducted using variable pressure mode at 15 keV. EDS spectroscopy was used to determine the changes in the oxygen to carbon ratio of the thermally treated UHMMPE fibers.

### 2.5. Free Radical Determination

Electron Paramagnetic Resonance (EPR) spectroscopy was used to evaluate the presence of free radicals in the thermally aged UHMMPE fibers. The measurements were carried out at room temperature in the presence of oxygen. The EPR spectra were acquired on a ESP300 spectrometer (Bruker Biospin, Billerica, MA, USA) using the following instrument parameters: microwave frequency of 9.43 GHz, microwave power of 6.35 mW, frequency modulation of 100 kHz, modulation amplitude of 6.23 G, receiver gain of 56,400, center field at 3350 G, a sweep width of 800 G, a conversion time of 20.48 ms, and a time constant of 20.48 ms. The instrument was calibrated prior to the measurements using a 2,2-diphenyl-1-picrylhydrazyl (DPPH) stable free-radical control sample. EPR measurements were conducted on both specimens aged according to Section 2.1, and on specimens specifically treated for the EPR experiments. UHMMPE fiber samples were prepared from unaged yarn. Specimens were heated to approximately 115 °C in air under an oil bath and were removed at specified intervals (approximately 30 min intervals for a total of 4 h) for EPR measurements. 

### 2.6. Chemical Characterization

Fourier Transform Infrared Spectroscopy (FTIR) was used to identify the changes in the chemical structure of the thermally aged polyethylene fibers. A Thermo Nicolet is 50 FTIR equipped with a Nicolet iTX Diamond attenuated total reflectance (ATR) accessory was used to obtain the IR spectra of the fiber samples (Thermo Fisher Scientific, Waltham, MA, USA). Bundle of fibers were tested, and all spectra were collected at a resolution of 8 cm^−1^ between 400 cm^−1^ and 4000 cm^−1^ wavenumbers. All spectra in this study represent an average of 128 scans. A background spectrum was collected and subtracted prior to each sample run. Typical standard uncertainties for spectral measurement are 8 cm^−1^ in wavenumber and 5% in peak intensity. FTIR-ATR measurements were performed on both samples aged according to Section 2.1 and the samples prepared for EPR according to the procedure described in Section 2.5.

## 3. Results and Discussion

### 3.1. Molar Mass of Thermally Aged Fibers

**Figure 1:**
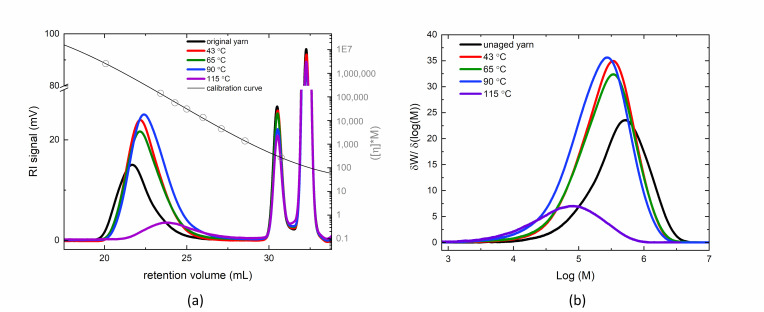
Representative chromatogram of the yarn unaged and under each aging condition with a calibration curve (**a**) and the resultant molar mass distribution of the PE fibers (**b**). The secondary peaks in the chromatogram (**a**) shown after 30 mL retention volume are the antioxidant and flow rate marker peaks, respectively. Note: All data associated with the figures in this paper have been archived and is publicly available through the following data publication: Amanda L. Forster (2021), Effects of Thermal Aging on Molar Mass of Ultra-High Molar Mass Polyethylene Fibers, Version 1.0.0, National Institute of Standards and Technology, https://doi.org/10.18434/mds2-2463, accessed on 23 November 2021.

The molar mass distributions (MMD) of the thermally aged fibers compared to the original, unaged fiber is summarized in Table 1, highlighting the first, second, and third moments of the molar mass distribution, as $\bar{M_{n}}$, $\bar{M_{w}}$, and $\bar{M_{z}}$, respectively. Upon aging conditions between nominally 43 °C and 115 °C, chain scission is observed by the decrease in $\bar{M_{n}}$, which is more pronounced with increasing aging temperature. Initially, it appears that longer polyethylene chains may undergo preferential scission at 43 °C conditions, as the dispersity, Ð, decreases by about 12.5% at that temperature. However, the application of additional heat, results in the fibers undergoing larger extent of chain scission across the MMD, resulting in shifts in the entire population of chains to lower molar mass and the broadening of the distribution, as shown in Figure 1. Furthermore, in the amorphous and highly oriented amorphous regions, we postulate that there is some crosslinking occurring of the higher molar mass chains in the 115 °C condition, as we observe lower chromatogram areas in the refractive index detector of the HT-SEC, even though all samples were dissolved in between 1 mg/mL to 2 mg/mL of TCB for injection. Although mass recovery is not a quantitative indicator of crosslinking, as one cannot assume that all injected chains elute from the column, the lower concentration observed in the 115 °C samples may indicate the presence of higher molar mass chains or networks, which may be less soluble, and fractionate out of solution or onto the column at the injection conditions.

### 3.2. Crystal Morphology and Crystallinity of Aged Fibers

**Figure 2:**
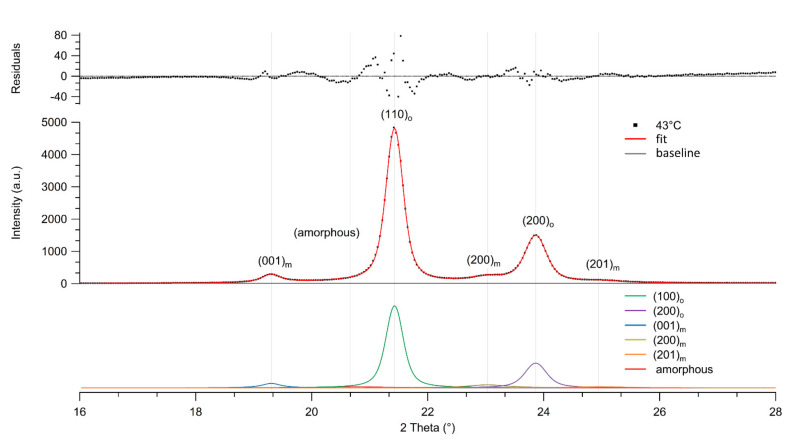
WAXS diffractogram of thermally aged UHMMPE fibers stored at nominally 43 °C for about 81 weeks. The resolved peaks for the orthorhombic and monoclinic crystals, as well as the amorphous halo are shown; the fit and residuals are also presented, and the chi squared value is *χ*^2^ = 1.084.

Wide angle X-ray analysis was performed to further the understanding of the morphological changes in the UHMMPE fibers due to thermal aging. Figure 2 shows the equatorial wide-angle X-ray scattering (WAXS) pattern from aged UHMMPE fibers stored at nominally 43 °C for about 21 weeks (w). It also includes the deconvolution of the diffraction peaks present in the spectrum along with the residuals of the fit using Voigt distributions, which are probability distributions given by a convolution of the Lorentz and Gaussian distributions. The WAXS patterns of unaged fibers and fibers aged at 43 °C for 81 w, 65 °C for 943 w, 90 °C for 17 w, and at 115 °C for 8 w, were deconvoluted and fitted using a procedure similar to the analysis by Russell et al. [40].

The results revealed the presence of two strong diffraction peaks at 2θ angles around 21.5° and 23.8° corresponding to the (110)_o_ and (200)_o_ lattice planes of orthorhombic crystals [40,41,42]. These were the strongest diffraction peaks, always present in the unaged and all thermally aged UHMMPE fibers tested in this study. Additionally, three weaker diffraction peaks present at 2θ angles around 19.5°, 23°, and 24.8° corresponding to the (001)_m_, (200)_m_, and (201)_m_ lattice planes of the monoclinic crystals within the fiber material [40,42] were observed. In addition, a very broad amorphous peak around a 2θ angle of 20.5° was consistently present in all the fibers tested [40,42]. As shown from the analysis of the WAXS data (Table 2), the percent amorphous phase and the total percent crystallinity remained constant for all fiber samples. However, the fibers aged at the highest temperature (115 °C) revealed a slight decrease in crystallinity after the material was kept for about 17 weeks at a temperature well above its alpha-relaxation, which has been previously reported at 80 °C [43]. At temperatures below and around the alpha-relaxation of the material, the crystallinity remains virtually unchanged. However, a decrease in the monoclinic crystal phase was observed as we transition from 43 °C to 65 °C and, finally, to 90 °C; indicating that the material undergoes a crystal phase transition from monoclinic to orthorhombic, as can be seen from the increase in orthorhombic to monoclinic ratio in Table 2. At 115 °C, very little monoclinic phase is still detected in the fibers. This is explained by the polyethylene monoclinic unit cell being less thermodynamically stable than the orthorhombic unit cell [30]. 

The monoclinic phase is typically found in fiber samples that were subjected to mechanical stress beyond the yield point, where this stress is either due to tension or compression [40], e.g., during the drawing process of the UHMMPE fibers where lateral compression is present [44]. In the case of our studies, a mechanical winder based on an electric drill was used to prepare the samples for the aging study, thereby subjecting the fibers to tensile and lateral stress, which could induce the formation of the monoclinic phase. Therefore, when the fibers are placed in the environmental chambers, the material relaxes this internal stress enthalpically through rotational motion of the C–C bond along the backbone of the UHMMPE chains, and this relaxation increases with increasing temperature. Bond rotation can lead to chain slippage and translational motion of the polymer molecules within the highly oriented region (or semi crystalline region) allowing the material to entropically relax due to the resultant change in the conformational freedom of the polymer chains [30]. This relaxation process could potentially result in the transformation of the monoclinic phase back to the more stable orthorhombic phase resulting in a system with higher entropy. This transition is more likely to happen in the interface of the two crystal phases, as the boundary between the (110)_o_ and (001)_m_ planes moves to the monoclinic side according to findings by Y. Takahashi et al. [45]. 

**Figure 3:**
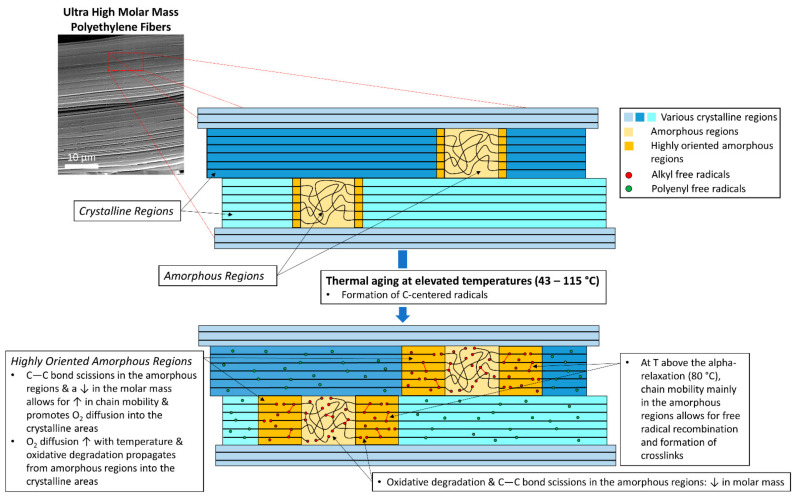
Thermal aging effects on ultra-high molar mass polyethylene fibers; C–C bond scissions, decrease in molar mass, and crosslinking.

Finally, these results demonstrate that at the temperatures used in this study, even those as high as 90 °C and 115 °C, the orthorhombic crystal network within the fibers is not significantly impacted and the minor loss in the total present crystallinity could be attributed to relaxation processes in the intermediate, oriented amorphous phase between the crystals [46]. The formation of crosslinks between C-centered radicals, primarily within the highly oriented amorphous parts of the fibers, is also possible, as depicted in Figure 3. This crosslinking-effect, likely played a role in maintaining the percent crystallinity of the material at the highest temperature aging conditions. In highly oriented, ultra-drawn UHMMPE fibers, it is difficult for oxygen to diffuse into the crystalline regions and induce further oxidation, more chain scission effects, and as a result, a reduction in crystallinity. 

### 3.3. Topography and Oxygen Content of Aged Fibers

**Figure 4:**
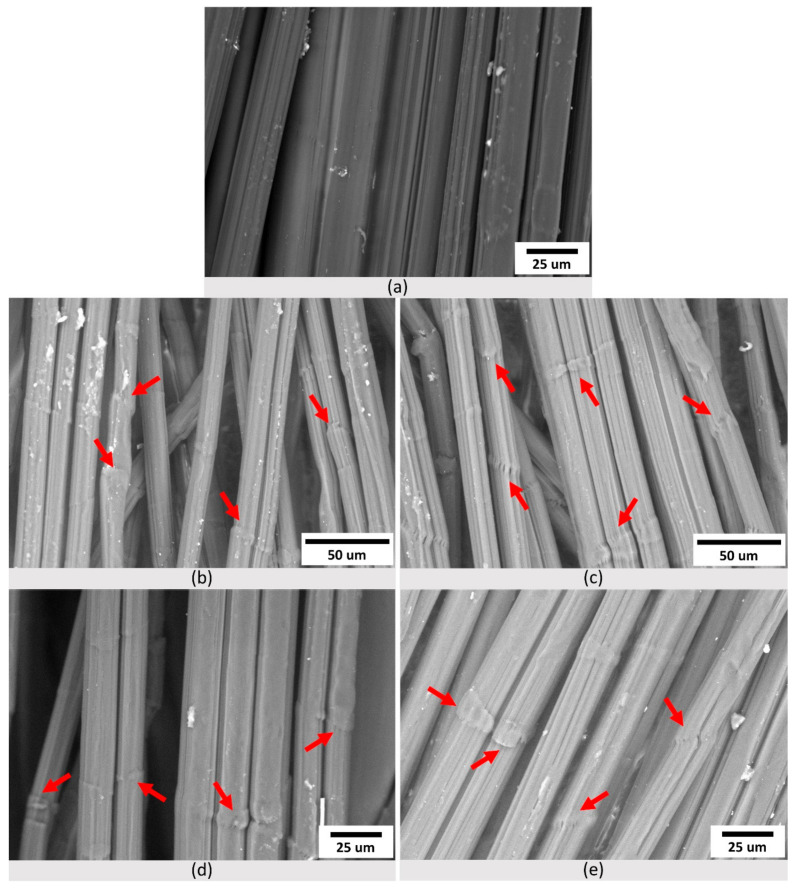
Scanning electron microscopy (SEM) micrographs of (**a**) control, and thermally aged UHMMPE fibers at (**b**) 43 °C, (**c**) 65 °C, (**d**) 90 °C, and (**e**) 115 °C, respectively.

All fibers were examined for evidence of morphological changes on their surface before and after the aging study. The SEM micrographs in Figure 4 confirm the fibrillar structure of the UHMMPE fibers, consisting of a large number of filaments easily distinguishable in the micrographs [47]. They also illustrate the surface damage and the formation of kink bands in the unaged and thermally aged UHMMPE fibers. Unaged fibers, as seen in Figure 4a, showed no evidence of kink bands since the material was not wrapped around the spools used for thermal aging. However, thermally aged fibers exhibited very similar morphological characteristics, at all temperatures, including the presence of kink bands (shown in red arrows, Figure 4). Kink bands are attributed to the mechanical stress applied during the bending and folding of the fibers around the spools used for aging [23].

The formation of kink bands due to bending and folding processes on UHMMPE fibers has been previously reported [23]. The kink bands are uniformly spread across the fibers of all thermally treated samples. In addition to the morphological examination, EDS elemental analysis was performed to evaluate the changes in the oxygen to carbon ratio within the material, specifically at the kink banded areas of the fibers. As expected, all aged fibers exhibited an increased oxygen to carbon ratio that was statistically significant (via a student *t*-test, *p* < 0.05) as compared to the unaged fibers (Table 3).

The increase in the O/C ratio can be attributed to several factors, including an increase in dissolved molecular oxygen within the fibers from the disruption of their fibrillar structure (e.g., kink bands), diffusion of oxygen in the amorphous regions of the material, and the formation of carbonyl groups due to the increasing thermal oxidation with increasing aging time. The trend of the increase in O/C ratio with aging temperature is consistent with previously published results of increasing oxidation index (OI) with aging temperature for this system, where the OI increased from essentially zero (0.0002) for unaged samples to 0.0471 (samples aged at about 115 °C for nominally 18 weeks) [16]. Based on the EDS data provided in this study, and the fact that the oxygen to carbon ratio remains nearly unchanged for all the aged samples at the kink banded regions shown with red arrows in Figure 4, it is proposed that molecular oxygen can diffuse into the fibers through mechanically induced structural defects (e.g., kink bands) and primarily saturates the amorphous regions of the material. Since there is a linear correlation between the solubility of oxygen in polyethylene and the amorphous volume fraction of the polymer, it is expected that the amount of dissolved oxygen will eventually reach an equilibrium, because the crystallinity of the material does not decrease significantly with aging. At the highest aging temperature (115 °C), which is above the polymer’s alpha relaxation (80 °C), oxygen diffusivity increases and could potentially promote the diffusion of oxygen molecules in the highly oriented amorphous regions, as shown in Figure 3.

### 3.4. The Presence of the Polyenyl C-Centered Free Radicals in Aged Fibers

Elevated temperatures during aging will promote thermal oxidation through thermally induced bond scissions, the formation of carbon-centered radicals, and ultimately, the formation of carbonyl groups in the polyethylene molecules via a complex cascade of reactions [23,48]. During thermal oxidation, the various alkyl macroradicals present primarily in the amorphous regions of the UHMMPE fibers will rapidly react with oxygen molecules nearby, diffuse into the amorphous areas of the fibers and form peroxyl radicals. Although peroxyl radicals are very stable in highly oriented UHMMPE fibers, a slight increase in temperature, of only 10 °C, can almost double the rate of hydrogen abstraction reaction from neighboring polymer chains to form new secondary alkyl carbon-centered macroradicals. The formation of peroxyl radicals and the hydrogen abstraction reaction are very important since they help propagate the thermo-oxidative degradation of UHMMPE, which can lead to C–C bond scission, as evidenced by the changes in molar mass presented in this study. The immediate result of C–C bond scission is the formation of alkyl carbon-centered radicals, which are unstable and can form crosslinks (intermolecular recombination), trans-vinylene unsaturation (intramolecular recombination), or can migrate to an allylic position to form allyl and/or polyenyl free radicals [29,49]. In the presence of oxygen, alkyl carbon centered radicals can rapidly react to form peroxyl and alkoxyl radicals primarily in the amorphous areas of the polymer, where oxygen diffusion is most favored [23].

**Figure 5:**
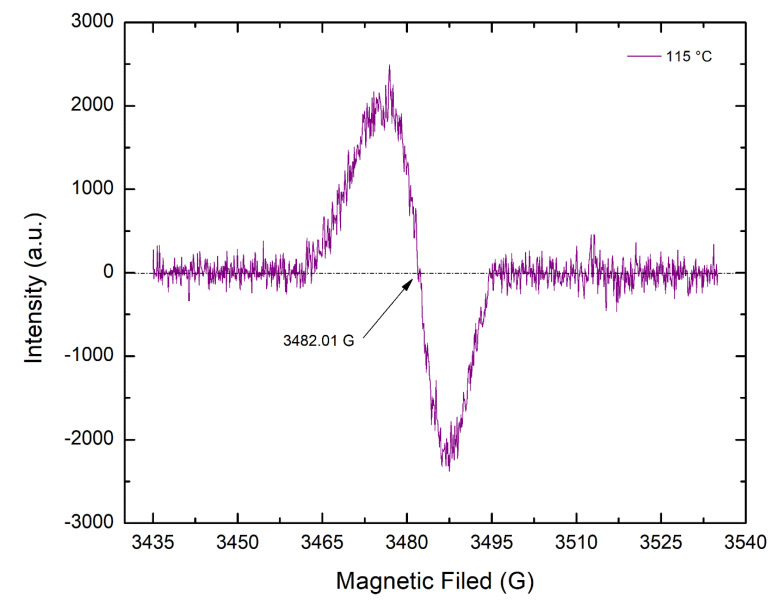
Electron paramagnetic resonance (EPR) spectrum of polyenyl free radicals detected in thermally aged UHMMPE fibers at 115 °C in air.

As previously mentioned, the UHMMPE fibers exhibit very high crystallinity, above 90%, as shown by the X-ray diffraction results, at all aging conditions. Thus, the mobility of the highly oriented polymer chains is reduced and the diffusion of oxygen in the fibers is limited only to the small amount of amorphous material present, minimizing the formation of peroxyl and alkoxyl radicals. Therefore, the alkyl carbon-centered radicals formed due to thermal degradation are expected to transform to mainly allyl or polyenyl free radicals within the crystalline areas of the polymer. This hypothesis is confirmed by EPR spectroscopy, where thermally aged UHMMPE fibers revealed a clear signal of a singlet at around 3482.01 G with a g-factor equal to 2.0050, which is very close to the values reported in the literature for polyenyl radicals [50]. Figure 5 shows a representative EPR spectrum for fibers aged at 115 °C in air. The presence of polyenyl radicals further supports the phenomenon of chain scission occurring due to thermal aging, mainly in the amorphous areas of the polymer, and the reduction in the polymer’s molar mass.

### 3.5. Unsaturations Present in Aged Fibers

**Figure 6:**
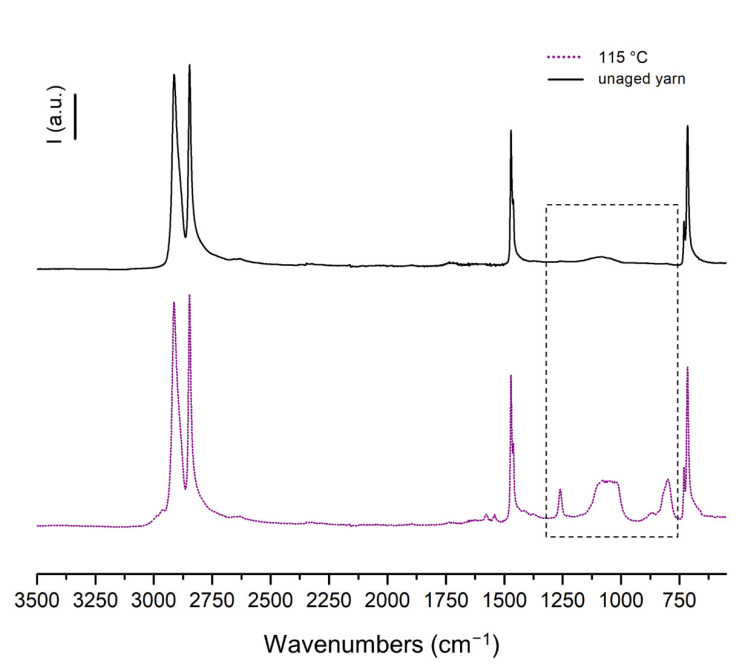
FTIR spectra of control unaged and thermally aged UHMMPE fibers at 115 °C in air; marked area highlights changes in the spectra after thermal aging due to presence of newly formed vinyl, trans-vinylene, alcohol, and ester groups.

The presence of polyenyl radicals in the aged UHMMPE fibers was also supported by molecular spectroscopy. Upon aging of the fibers at 115 °C in air, it was clear that new bands were formed in the region nominally between 800 cm^−1^ to 1300 cm^−1^. Within this region, the infrared absorption bands can be assigned to various vibrational modes of vinyl and trans-vinylene groups (800 cm^−1^ to 1000 cm^−1^), as well as some alcohols (1096 cm^−1^) and esters (1264 cm^−1^) [51]. The shoulder peak at around 2900–3000 cm^−1^ is associated with the presence of methyl bands in polyethylene. These shoulder peaks are typical in the IR spectrum of polyethylene, because as the crystallinity or the crystal structure of PE changes, the various methyl groups could result in slight changes in the methylene sp^3^ C–H asymmetric and symmetric stretching bands (at 2916 cm^−1^ and 2848 cm^−1^). The peaks in the region between 1500 and 1650 cm^−1^ could be associated and further support the presence of unsaturations (C=C) in the PE fibers. The formation of these groups is related to thermal degradation via free radical formation and thermal oxidation, which can also lead to crosslinking during aging, as previously discussed [23,49]. Unsaturations are mainly formed in the highly oriented regions of the polymer, where oxygen diffusion is minimal. Under these conditions, C-centered radicals can undergo disproportionation reaction leading to the formation of double bonds along the backbone of the polymer. However, in the amorphous regions, where oxygen molecules are present, C-centered radicals react with oxygen and leading to thermo-oxidative degradation and ultimately, the formation of alcohols and esters. Figure 6 below, shows two representative spectra for the unaged and thermally aged UHMMPE fibers at 115 °C.

## 4. Conclusions

The HT-SEC results demonstrate that when exposed to temperatures from 43 °C to 115 °C or periods of time up to 2 years, UHMMPE fibers showed a reduction in number average ($\bar{M_{n}}$) molar mass of approximately 92% due to thermally induced rupture of the C–C bonds. Ruptures of the C–C bonds are expected to occur primarily in the crystalline regions, which are brittle from the mechanical stress of the ultra-drawing process used during fiber manufacture. These C–C bond scissions in the backbone of the polymer can result in the formation of primary alkyl carbon centered radicals. Furthermore, the C–C scission can be produced through oxidation reactions. This was confirmed by the presence of oxygen containing groups in the molecular analysis after aging, which is indicative of the material’s oxidation. Thermal oxidation is predominantly present in the amorphous and highly oriented amorphous areas of the polymer, where oxygen can diffuse into. The WAXS data in this study have shown a significant increase in the amorphous areas of the polyethylene fibers from around 3.1% in the unaged material to about 12.5% at 115 °C. This evidence further supports the occurrence of thermo-oxidative degradation and bond ruptures, which ultimately result in a reduction in the molar mass, as shown by the HT-SEC measurements. A significant decrease in the molar mass was observed from the HT-SEC measurements of the fibers aged at 115 °C. However, it is important to mention that the concentration of this sample during the HT-SEC measurements was significantly lower than that of the rest of the samples. This can be attributed to the polymer’s inability to fully dissolve because potential crosslinking in the amorphous regions may have caused partial gelation of the sample, as depicted in Figure 3. Additionally, EPR measurements detected the formation of polyenyl radicals in the samples aged at 115 °C in air, which were further supported by molecular spectroscopy showing the formation of unsaturations in these specimens that were not observed in the unaged specimens. The formation of polyenyl radicals is attributed to the thermal aging of the material and has been previously shown to form after the formation of secondary alkyl radicals in the backbone of polyethylene via the abstraction of H-atoms by the alpha-carbon centered radicals. These radicals are known to participate in very fast intermolecular reactions, which are thermodynamically favored with a ΔH = −288 kJ/mol, to produce vinylene double bonds, as shown in the IR spectrum, and molecular hydrogen [52,53]. Polyenyl radicals are mainly known to be present in the crystalline areas of the fibers where oxygen cannot easily penetrate to induce oxidation and can survive for an extended period of time (years) at room temperature [29,54]. These findings support the idea that thermal degradation, oxidation, and chain scission effects are primarily present in the amorphous and highly oriented amorphous regions of the fibers. Overall, this work has shown that thermal aging, especially at temperatures above the alpha-relaxation of polyethylene, results in oxidation, chain scission, and the formation of unsaturations and polyenyl radicals in the material. We have previously shown that UHMMPE fibers are expected to perform well at use conditions for body armor (near body temperature). However, the above factors should be considered when using these fibers in conditions in which they might be exposed to elevated temperatures for long periods of time.

## Figures and Tables

**Table 1:** Molar mass averages representing the number, mass, and z-average molar masses ($\bar{M_{n}}$, $\bar{M_{w}}$, and $\bar{M_{z}}$, respectively) and dispersity (Ð = $\bar{M_{w}}$ /$\bar{M_{n}}$). Data presented are mean ± standard error of the mean (S.E.M.), where *n* ≥ 3.

Sample	Mn (× 10−5) (g/mol)	Mw (× 10−5) (g/mol)	Mz (× 10−5) (g/mol)	Ð
Unaged	2.31 ± 0.88	6.53 ± 1.04	9.69 ± 1.30	3.2 ± 1.2
43 °C, 81 w	2.02 ± 0.40	5.66 ± 1.25	8.91 ± 1.89	2.8 ± 0.4
65 °C, 43 w	1.74 ± 0.21	5.95 ± 0.37	10.3 ± 0.31	3.5 ± 0.6
90 °C, 17 w	1.02 ± 0.17	5.12 ± 0.38	9.34 ± 0.80	5.2 ± 1.0
115 °C, 8 w	0.19 ± 0.10	1.52 ± 0.49	4.94 ± 1.97	8.0 ± 3.4

**Table 2:** Summary of the estimates of the crystallinity and crystal phase percentages for the orthorhombic and monoclinic crystals present in the control and the thermally aged UHMMPE fibers as calculated from the fitting of WAXS data.

Thermal Treatment	Amorphous (%)	Orthorhombic Crystals (%)		Monoclinic Crystals (%)			Ortho/Mono Ratio	Crystallinity WAXS (%)
		(110)	(200)	(010)	(200)	(210)		
Unaged	3.1	68.3	23.6	2.0	2.8	0.2	18.5	96.9
43 °C, 81 w	3.7	60.1	23.1	4.3	5.9	2.9	6.3	96.3
65 °C, 43 w	2.8	63.1	25.7	2.2	4.4	1.8	10.5	97.2
90 °C, 17 w	2.4	65.3	27.5	0.9	3.9	-	19.0	97.6
115 °C, 8 w	12.5	61.5	23.6	0.6	1.8	-	34.8	87.5

**Table 3:** Changes in the oxygen to carbon ratio with thermal aging of UHMMPE fibers, specific to the kink-banded areas, based on SEM-EDS study and the atomic percentage data of each element. Data presented are mean ± S.E.M. (*n* ≥ 20).

Thermal Treatment	Oxygen (At. %)	Carbon (At. %)	O/C Ratio
Unaged	2.93 ± 0.20	97.07 ± 0.20	0.03
65 °C, 43 w	6.64 ± 0.12	93.37 ± 0.12	0.07
90 °C, 17 w	6.27 ± 0.23	93.74 ± 0.23	0.07
115 °C, 8 w	7.59 ± 0.31	92.33 ± 0.27	0.08

## Data Availability

Data associated with this article is freely and publicly available through the NIST Public Data Repository. It is published as “Effects of Thermal Aging on Molar Mass of Ultra-High Molar Mass Polyethylene Fibers” Identifier: doi:10.18434/mds2-2463, accessible at web address: https://doi.org/10.18434/mds2-2463, accessed on 23 November 2021.

## References

[1] Folgar F. (2016). Thermoplastic matrix combat helmet with carbon-epoxy skin for ballistic performance. Advanced Fibrous Composite Materials for Ballistic Protection.

[2] Wittmann J., Lotz B. (1989). Epitaxial crystallization of monoclinic and orthorhombic polyethylene phases. Polymer.

[3] Folgar F. (2016). Ballistic Helmets and Method of Manufacture Thereof. U.S. Patent.

[4] Mazelsky B. (1999). Flexible Body Armor. U.S. Patent.

[5] Joseph A., Wiley A., Orr R., Schram B., Dawes J.J. (2018). The impact of load carriage on measures of power and agility in tactical occupations: A critical review. Int. J. Environ. Res. Public Health.

[6] Schram B., Orr R., Pope R., Hinton B., Norris G. (2018). Comparing the effects of different body armor systems on the occupational performance of police officers. Int. J. Environ. Res. Public Health.

[7] Crouch I.G. (2019). Body armour—New materials, new systems. Def. Technol..

[8] DSM Dyneema Tech (2016). Ultra High Molecular Weight Polyethylene Fiber from DSM Dyneema. DataSheet. https://extreemasoftslings.com/wp-content/uploads/2019/02/Dyneema-UHMWPF.pdf.

[9] Sanborn B., DiLeonardi A.M., Weerasooriya T. (2015). Tensile properties of Dyneema SK76 single fibers at multiple loading rates using a direct gripping method. J. Dyn. Behav. Mater..

[10] James N. (2016). Body Armor for Law Enforcement Officers: In Brief. https://ecommons.cornell.edu/bitstream/handle/1813/79529/CRS_Body_Armor_for_Law_Enforcement.pdf?sequence=1&isAllowed=y.

[11] Chin J., Forster A., Rice K. (2009). Chapter 10: Field and Laboratory Aging Effects on Poly (*p*-phenylene benzobisoxazole) Fibers Used in Body Armor. Polymer Degradation and Performance.

[12] Hart S.V. (2004). Report to the Attorney General on Body Armor Safety Initiative Testing and Activities.

[13] Office of Justice Programs, US Department of Justice (2005). Third Status Report to the Attorney General on Body Armor Safety Initiative Testing and Activities.

[14] Messin G.H., Rice K.D., Riley M.A., Watson S.S., Sieber J.R., Forster A.L. (2011). Effect of moisture on copolymer fibers based on 5-amino-2-(p-aminophenyl)-benzimidazole. Polym. Degrad. Stab..

[15] Forster A.L., Pintus P., Messin G.H., Riley M.A., Petit S., Rossiter W., Chin J., Rice K.D. (2011). Hydrolytic stability of polybenzobisoxazole and polyterephthalamide body armor. Polym. Degrad. Stab..

[16] Forster A.L., Forster A.M., Chin J.W., Peng J.S., Lin C.C., Petit S., Kang K.L., Paulter N., Riley M.A., Rice K.D. (2015). Long-term stability of UHMWPE fibers. Polym. Degrad. Stab..

[17] Costa L., Luda M., Trossarelli L. (1997). Ultra-high molecular weight polyethylene: I. Mechano-oxidative degradation. Polym. Degrad. Stab..

[18] Costa L., Luda M., Trossarelli L. (1997). Ultra high molecular weight polyethylene—II. Thermal-and photo-oxidation. Polym. Degrad. Stab..

[19] Costa L., Bracco P. (2016). Mechanisms of cross-linking, oxidative degradation, and stabilization of UHMWPE. UHMWPE Biomaterials Handbook.

[20] Lacoste J., Carlsson D. (1992). Gamma-, photo-, and thermally-initiated oxidation of linear low density polyethylene: A quantitative comparison of oxidation products. J. Polym. Sci. A Polym. Chem..

[21] Lacoste J., Deslandes Y., Black P., Carlsson D. (1995). Surface and bulk analyses of the oxidation of polyolefins. Polym. Degrad. Stab..

[22] Chin J.W., Byrd E., Clerici C., Forster A.L., Oudina M., Sung L.P., Rice K.D. (2006). Chemical and Physical Characterization of Poly(*p*-phenylene-2,6-benzobisoxazole) Fibers Used in Body Armor: Temperature and Humidity Aging.

[23] Tsinas Z., Forster A.L., Al-Sheikhly M. (2018). Oxidation reactions in kink banded regions of UHMMPE fiber-based laminates used in body armor: A mechanistic study. Polym. Degrad. Stab..

[24] Dijkstra D., Pennings A. (1988). The role of taut tie molecules on the mechanical properties of gel-spun UHMWPE fibres. Polym. Bull..

[25] Klein P., Woods D., Ward I. (1987). The effect of electron irradiation on the structure and mechanical properties of highly drawn polyethylene fibers. J. Polym. Sci. B Polym. Phys..

[26] Wilding M.A., Ward I.M. (1981). Routes to improved creep behaviour in drawn linear polyethylene. Plast. Rubber Process. Appl..

[27] Deng M., Latour R., Drews M., Shalaby S.W. (1996). Effects of gamma irradiation, irradiation environment, and postirradiation aging on thermal and tensile properties of ultrahigh molecular weight polyethylene fibers. J. Appl. Polym. Sci..

[28] De Boer J., Pennings A. (1981). Crosslinking of ultra-high strength polyethylene fibers by means of γ-radiation. Polym. Bull..

[29] Forster A.L., Tsinas Z., Al-Sheikhly M. (2019). Effect of irradiation and detection of long-lived polyenyl radicals in highly crystalline ultra-high molar mass polyethylene (UHMMPE) fibers. Polymers.

[30] Kerber R.W. (1983). Schnabel: *Polymer Degradation, Principles and Practical Applications*. Carl Hanser Verlag, München 1981. 227 Seiten, Preis: DM 68,—. Ber. Bunsenges. Phys. Chem..

[31] Zhao L., Song P.a., Cao Z., Fang Z., Guo Z. (2012). Thermal stability and rheological behaviors of high-density polyethylene/fullerene nanocomposites. J. Nanomater..

[32] Nyden M.R., Stoliarov S.I., Westmoreland P.R., Guo Z., Jee C. (2004). Applications of reactive molecular dynamics to the study of the thermal decomposition of polymers and nanoscale structures. Mater. Sci. Eng. A.

[33] Stoliarov S.I., Lyon R.E., Nyden M.R. (2004). A reactive molecular dynamics model of thermal decomposition in polymers. II. Polyisobutylene. Polymer.

[34] Knyazev V.D. (2007). Effects of Chain Length on the Rates of C−C Bond Dissociation in Linear Alkanes and Polyethylene. J. Phys. Chem. A.

[35] Chabba S., Van Es M., Van Klinken E., Jongedijk M., Vanek D., Gijsman P., Van Der Waals A. (2007). Accelerated aging study of ultra high molecular weight polyethylene yarn and unidirectional composites for ballistic applications. J. Mater. Sci..

[36] Jung M.R., Balazs G.H., Work T.M., Jones T.T., Orski S.V., Rodriguez C.V., Beers K.L., Brignac K.C., Hyrenbach K.D., Jensen B.A. (2018). Polymer identification of plastic debris ingested by pelagic-phase sea turtles in the central Pacific. Environ. Sci. Technol..

[37] Jung M.R., Horgen F.D., Orski S.V., Rodriguez V., Beers K.L., Balazs G.H., Jones T.T., Work T.M., Brignac K.C., Royer S.-J. (2018). Validation of ATR FT-IR to identify polymers of plastic marine debris, including those ingested by marine organisms. Mar. Pollut. Bull..

[38] (2012). Standard Test Method for Determining Molecular Weight Distribution and Molecular Weight Averages of Polyolefins by High Temperature Gel Permeation Chromatography.

[39] Roe R.-J., Roe R. (2000). Methods of X-ray and Neutron Scattering in Polymer Science.

[40] Russell K., Hunter B., Heyding R. (1997). Monoclinic polyethylene revisited. Polymer.

[41] Kwon Y., Boller A., Pyda M., Wunderlich B. (2000). Melting and heat capacity of gel-spun, ultra-high molar mass polyethylene fibers. Polymer.

[42] Joo Y., Han O., Lee H.-K., Song J. (2000). Characterization of ultra high molecular weight polyethyelene nascent reactor powders by X-ray diffraction and solid state NMR. Polymer.

[43] Stadler F.J., Kaschta J., Münstedt H. (2005). Dynamic-mechanical behavior of polyethylenes and ethene-/α-olefin-copolymers. Part I. α′-Relaxation. Polymer.

[44] Yeh J.-T., Lin S.-C., Tu C.-W., Hsie K.-H., Chang F.-C. (2008). Investigation of the drawing mechanism of UHMWPE fibers. J. Mater. Sci..

[45] Takahashi Y., Ishida T., Furusaka M. (1988). Monoclinic-to-orthorhombic transformation in polyethylene. J. Polym. Sci. B Polym. Phys..

[46] Hu W., Buzin A., Lin J.S., Wunderlich B. (2003). Annealing behavior of gel-spun polyethylene fibers at temperatures lower than needed for significant shrinkage. J. Polym. Sci. B Polym. Phys..

[47] Berger L., Kausch H., Plummer C. (2003). Structure and deformation mechanisms in UHMWPE-fibres. Polymer.

[48] Li C.-S., Zhan M.-S., Huang X.-C., Zhou H. (2012). Degradation behavior of ultra-high molecular weight polyethylene fibers under artificial accelerated weathering. Polym. Test..

[49] Tsinas Z. (2016). Towards an Understanding of the Degradation Mechanisms of UHMWPE-Based Soft Ballistic Inserts. Ph.D. Thesis.

[50] Kasser M.J., Silverman J., Al-Sheikhly M. (2010). EPR simulation of polyenyl radicals in ultrahigh molecular weight polyethylene. Macromolecules.

[51] Tantipattarakul S., Vaughan A.S., Andritsch T. (2020). Ageing behaviour of a polyethylene blend: Influence of chemical defects and morphology on charge transport. High Volt..

[52] Johnson W., Lyons B. (1995). Radiolytic formation and decay of trans-vinylene unsaturation in polyethylene: Fourier transform infra-red measurements. Radiat. Phys. Chem..

[53] Dole M., Milner D., Williams T.F. (1958). Irradiation of polyethylene. II. Kinetics of unsaturation effects. J. Am. Chem. Soc..

[54] Jahan M.S. (2009). ESR insights into macroradicals in UHMWPE. UHMWPE Biomaterials Handbook.

